# The Therapeutic Potential of Myo-Inositol in Managing Patients with Respiratory Diseases

**DOI:** 10.3390/ijms26052185

**Published:** 2025-02-28

**Authors:** Cristina Quecchia, Andrea Vianello

**Affiliations:** 1 Pediatric Allergy Service, Children’s Hospital, ASST Spedali Civili di Brescia, 25123 Brescia, Italy; quecchia@gmail.com; 2 Department of Cardiac, Thoracic, Vascular Sciences and Public Health, University of Padova, 35128 Padova, Italy

**Keywords:** myo-inositol, respiratory diseases, mucociliary clearance, pulmonary surfactant

## Abstract

Respiratory diseases are major health concerns worldwide. Chronic respiratory diseases (CRDs) are the third leading cause of death worldwide and some of the most common are chronic obstructive pulmonary disease (COPD), asthma, occupational lung diseases, and pulmonary hypertension. Despite having different etiology and characteristics, these diseases share several features, such as a persistent inflammatory state, chronic oxidative stress, impaired mucociliary clearance, and increased alveolar surface tension. CRDs are not curable; however, various forms of treatment, that help restore airway patency and reduce shortness of breath, can improve daily life for people living with these conditions. In this regard myo-inositol may represent a valid therapeutic adjuvant approach due to its properties. Being a redox balancer, an inflammation modulator, and, most importantly, a component of pulmonary surfactant, it may improve lung function and counteract symptoms associated with respiratory diseases, as recently evidenced in patients with COPD, COVID-19, asthma, and bronchiectasis. The aim of this review is to evaluate the potential therapeutic role of myo-inositol supplementation in the management of patients with respiratory diseases.

## 1. Introduction

Respiratory diseases represent a wide range of pathological conditions that can vary from mild and self-limiting conditions to life-threatening and persistent diseases [1]. Airway diseases affect both upper and lower tracts of the respiratory system and can be the consequences of complex gene–environment interactions, such as asthma, or caused by different etiological agents [2]. Respiratory diseases have been increasing in recent years, in particular, the recent COVID-19 pandemic has led to an unprecedented surge in respiratory problems, with an extraordinary increase in clinical manifestations, ranging from atypical pneumonia to acute respiratory distress syndrome [3]. Chronic respiratory diseases (CRDs) are the third leading cause of death worldwide, affecting more than 545 million people globally [4]. Chronic obstructive pulmonary disease (COPD) and asthma are the most prevalent CRDs, with global prevalences of 3.9% and 3.6%, respectively [5]. Despite being a serious health and economic burden, respiratory diseases have been proportionately receiving less public attention and less research funding than other diseases, such as cardiovascular disease, cancer, stroke, diabetes, and Alzheimer’s disease [6,7]. All of them are characterized by different etiology but may share common features, such as a significant alteration in mucociliary clearance, altered pulmonary surfactant homeostasis, persistent inflammation, and chronic oxidative stress [8,9]. An inflammatory state in the upper respiratory tract can have a negative impact on the lower, and so on. An alteration of the lung surfactant can negatively affect not only the health of the lungs but also that of the entire respiratory system, thus contributing to several inflammatory diseases’ progression and symptoms [9].

This is consistent with the concept of “unified airway disease” (UAD) that has been increasingly recognized in recent years. According to this concept, upper and lower airways form a single organ, with upper and lower airway diseases frequently co-occurring because they reflect different epidemiological, pathophysiological, and clinical manifestations of a single underlying disease process. The nasopharynx and the lungs, in addition to being joined from an anatomical point of view, share a common pathophysiological and immunological basis that supports the concept of “one airway, one disease” [10]. Supporting this concept, scientific evidence has shown, for example, that 40% of patients with allergic rhinitis suffer from asthma, while 90% of patients with asthma also suffer from allergic rhinitis [11]. Moreover, the severity of rhinitis is positively correlated with the score of asthma severity and inversely correlated to the index of quality of life. Moreover, patients with severe uncontrolled asthma commonly have severe nasal diseases [12].

The archetypal unified airway disease is asthma–rhinitis, but UAD is complex and heterogenous and may present other clinical phenotypes including lower airway disease, e.g., bronchiectasis and COPD [13].

The link between upper and lower airways is also reflected in therapeutic practice. For example, it has been shown that careful allergic rhinitis management is associated with better asthma control and, likewise, the improvement of asthma was associated with a resolution of allergic rhinitis symptoms [14]. Considering the prevalence and the impact on general health, prevention, control, and promotion of respiratory health should be an absolute priority in global decision making and action in the healthcare area. Therefore, scientific research should always look for new treatments and adjuvant therapies promoting respiratory health.

Moreover, by considering the concept of “unified airway disease”, the main goal for clinical research would be to use adjuvant therapies able to act simultaneously at different levels.

Our hypothesis is that, in this global scenario, myo-inositol (Myo-Ins) could represent a “non-pharmacological” adjuvant therapeutic strategy for the treatment of different airway and pulmonary diseases. Myo-Ins is a pleiotropic molecule participating in several intracellular signaling pathways and physiological processes. Moreover, considering its properties as redox balancer, inflammation modulator, and, most importantly, component of pulmonary surfactant, we believe that supplementation with Myo-Ins may have a direct impact on pulmonary system health. In fact, targeting several pathological processes is likely to lead to clinical improvement in both halves of the respiratory tract. The aim of this review is to highlight the properties of Myo-Ins by evidencing its potential role in counteracting pulmonary diseases, relieving symptoms, and preventing respiratory disease complications.

## 2. Myo-Inositol (Myo-Ins)

Myo-inositol (Myo-Ins) is a naturally occurring sugar derivative found in most foods including fruits, beans, and breast milk. It belongs to the “inositol family” that represents the most common stereoisomers in nature, playing major roles in a variety of metabolic pathways [15].

Endogenous Myo-Ins can comes from the breakdown of glucose and the conversion of D-glucose-6-phosphate, as well as through recycling of cellular phosphatidylinositol. Its phosphorylated derivatives comprise a variety of phosphoinositide stereoisomers, including inositol-1-4-5-trisphosphate (IP3) and diacylglycerol (DAG), which act as second messengers regulating Ca^2+^ signaling and PKC activity, respectively.

The biosynthesis of Myo-Ins requires nicotinamide adenine dinucleotide+ and magnesium. Thus, a deficiency in either may lead to a deficiency in Myo-Ins [16]. For example, Mg^2+^ deficiency can lead to exacerbations of pulmonary diseases as it helps in alleviating bronchospasm [17]. Moreover, low levels of serum Mg^2+^ are correlated with acute exacerbation of COPD, since hypomagnesemia is one of the common findings in patients with COPD, affecting patients with stage 3 and stage 4 COPD [18,19]. In general, it is well known that abnormalities in Myo-Ins metabolism are implicated in the pathophysiology of a variety of clinical settings including diabetic neuropathy, gestational diabetes, polycystic ovary syndrome (PCOS), bronchopulmonary, dysplasia, and acute respiratory distress syndrome.

Myo-Ins can either be present in free form or bound to phospholipids or inositol phosphate derivates. Most importantly, Myo-Ins is a structural component of the cell membrane as it is needed in the formation of phosphatidylinositol. It mediates signal transduction in response to several hormones, neurotransmitters, and growth factors and participates in osmoregulation. Biologically there is a high abundance of Myo-Ins in a variety of different tissues throughout the body. Millimolar concentrations of Myo-Ins have been detected, for example, within brain neurons [20]. Since there is a higher brain concentration of Myo-Ins compared to the whole-body levels at birth, it is not surprising that Myo-Ins may play a critical role in the development of the respiratory control system [21].

Being a pleiotropic molecule and generally recognized as safe (GRAS) by the Food and Drug Administration (FDA), Myo-Ins supplementation is applicable in different therapeutic areas and for long periods without adverse events.

Myo-Ins is also a second messenger of insulin and often used as a dietary supplement for women with gestational diabetes [22].

Myo-Ins has, for example, a pivotal role in several cellular pathways directly connected with reproductive functions by acting as a follicle-stimulating hormone (FSH) second messenger and activating different pathways that regulate the proliferation and maturation of granulosa cells [23].

Myo-Ins is successfully used to treat patients with polycystic ovary syndrome (PCOS), the most common reproductive endocrine disease. PCOS is characterized by metabolic abnormalities and increased expression of androgens that lead to exacerbation of acne, hirsutism, and alopecia and may impair women’s health status and quality of life [24]. Interestingly, a high prevalence of irregular menstrual cycles, infertility, obesity, and insulin resistance is also reported in asthmatic women. Patients with PCOS have higher probability of developing asthma compared to the general female population [25,26]. It seems that PCOS and asthma are connected. Asthma is one of the most common chronic respiratory diseases, with over 43 million new cases worldwide each year [27]. Asthmatic patients have recurrent or episodic wheezing, shortness of breath, chest tightness, and cough, mainly occurring during nighttime or early in the morning, after exercise, because of exposure to allergens/cold air, or after intake of medications such as acetylsalicylic acid or blockers. Moreover, asthmatic patients have multiple comorbidities including rhinitis, sinusitis, airway infections, obesity sleep-related breathing disorders, and PCOS. Therefore, due to several common features between asthma and PCOS, some authors proposed the definition of “asthma–PCOS overlap syndrome” to indicate a medical condition which shares characteristics of both diseases [28]. Since oral Myo-Ins is effective in improving symptoms and quality of life in women with PCOS, preliminary results support the hypothesis that administration of Myo-Ins may also be beneficial in patients with asthma [28].

## 3. Myo-Inositol and Respiratory System

### 3.1. Myo-Inositol and Mucociliary Clearance

The human airway is lined by a ciliated cylindrical pseudostratified epithelium and a layer of mucus, which is produced by submucosal glands and goblet cells scattered over the epithelium [29]. This integrated system of cilia and mucus characterizes mucociliary clearance (MCC), an innate lung defense mechanism from the terminal bronchiole to the larynx that protects the airway system from the harmful consequences of inhaled agents, including those of biological, chemical, and physical nature [30]. In healthy individuals, an effective MCC system coordinates the mucus formation that traps the inhaled particles and clears them through ciliary movement [29].

MCC ensures the cleaning of nasal cavities and paranasal sinuses and avoids the occurrence of infections. Virtually, mucus dysfunction occurs in all inflammatory airway diseases: the malfunctioning of one or more components of the MCC apparatus contributes to mucus accumulation, a classic clinical problem of many airway diseases [31].

Therefore, disorders affecting mucus quantity, quality, and/or ciliary dysfunction may lead to impaired MCC and ultimately to obstruction and inflammation of small airways, thus increasing the risk of respiratory infections, lung injuries, lung repair problems, chronic dysfunctions, and progression of respiratory diseases. Mucociliary clearance impairment has also been evidenced, in patients with respiratory syndrome coronavirus 2 (SARS-CoV-2) and dyspnea [32]. In chronic respiratory diseases, the persistence of airway inflammation can determine epithelial pathophysiologic modifications, thus leading to excessive mucus hypersecretion with abnormal mucus consistency, causing airway obstruction [33]. A decrease in MCC and the degree of its impairment correlates with the severity of several respiratory diseases [34,35]. Mucus hypersecretion occurs in response to recurrent infections and persistent inflammation, which worsen the physical properties and the clearance of the mucus [36]. Mucociliary clearance is impaired in COPD patients. COPD is a major cause of morbidity and mortality worldwide and comprises multiple components which, as well as a systemic component, include pulmonary inflammation, airway remodeling, and mucociliary dysfunction. The latter features contribute to the development of chronic, progressive airflow limitation. The mucociliary dysfunction component of COPD is due to mucus hypersecretion coupled with a decrease in mucus transport and represents an important pathophysiological feature requiring appropriate treatment [37]. 

Many functional studies in asthma patients have evidenced impaired mucociliary clearance and abnormal clearance of secretions, too [30,31,38]. Also, in acute and chronic infections, the immune inflammatory response to infection can alter airway cilia function, impair MCC, and lead to retained secretions [34].

Moreover, MCC is altered in patients with laryngeal and nasopharyngeal cancer undergoing radiation therapy [35]: in these patients, radiotherapy induces a prolonged and severe MCC impairment, thus inducing an abnormal biophysical transformation of airways mucus, which results in being highly adhesive and sticky [35].

**Figure 1:**
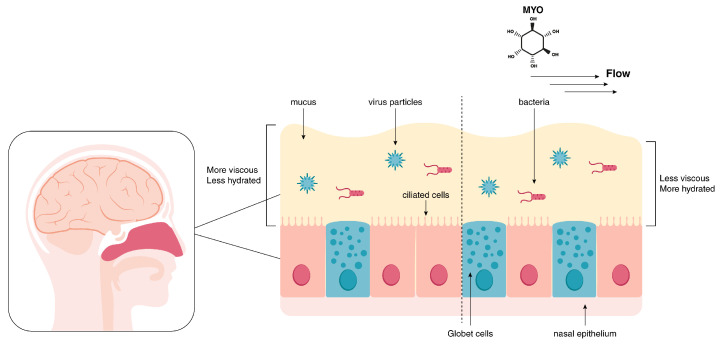
Mucociliary clearance. The nasal mucosa traps particles, including pathogens (bacteria and virus particles), to prevent their entry further into the respiratory tract. Cilia movement propels the mucus directionally, away from the cell surface. Myo-inositol (MYO), being a major intracellular osmolyte, may recruit water, thus increasing hydration of mucus and favoring mucociliary clearance.

Recently, a great interest in finding effective ways to directly stimulate MCC has led to several different new therapies with the aim of promoting airway surface hydration [39,40]. These include inhalation of aerosols of hypertonic saline or dry powder formulation for promoting the flux of water across the lung surface. In this regard, Myo-Ins may represent a valid therapeutic adjuvant approach. Myo-Ins is a powerful osmolyte in different tissues, such as the brain and kidney medulla [41]. Due to its biological functions, it can bind and retain large amounts of water. Its lubricating and emollient properties may alleviate inflammation-related symptoms, thus promoting MCC [42] (Figure 1).

In this regard, an interesting case emerged in bronchiectasis patients. Bronchiectasis is defined radiographically by permanent dilatation of the bronchi and clinically by cough, sputum production, and recurrent chest infections. Recognized as a major clinical problem since its first description by the French physician René Laennec in 1819 [43], bronchiectasis syndrome has a prevalence of 566 cases per 100,000 inhabitants [44].

It represents the final common pathway of several diseases. For instance, COPD appears to be a very common etiology, with bronchiectasis reported in up to 50% of patients with moderate-to-severe COPD [45]. Bronchiectasis also appears to be relatively common in patients meeting diagnostic criteria for severe asthma [46]. Also, gastro-esophageal reflux frequently co-exists with bronchiectasis and has been suggested as an etiological factor in some cases [47].

Bronchiectasis is a heterogeneous disease with a highly variable impact on patients. Severity ranges from patients without daily symptoms who have infrequent exacerbations to patients requiring lung transplantation.

The main symptom of bronchiectasis is excess mucus production, which can make the lungs more vulnerable to infection and increase the possibility of developing other symptoms such as cough, chest discomfort, and weight loss. Moreover, the literature indicates the involvement in the complex pathogenesis of bronchiectasis of an impaired MCC. As a consequence, patients with bronchiectasis have low lung functionality and a higher predisposition to bacterial colonization compared to the healthy population. Mucus in bronchiectasis patients has higher mucin concentrations and thus a higher percentage of solids, higher osmotic pressure, and increased elasticity and viscosity [48]. Furthermore, the abnormal mucus properties result in local hypoxia at the bronchial mucosa which further incites inflammation, increases mucin concentration, and worsens mucus properties [49].

Among treatments used to counteract bronchiectasis, inhaled antibiotics the most commonly used. They are used to reduce the bacterial load, and can increase the local and systemic inflammatory response. An updated Cochrane review of seven randomized controlled trials in adults with bronchiectasis found that inhaled corticosteroids, did not improve lung function or exacerbation frequency in patients with bronchiectasis [50].

Recently, a retrospective study analyzed for the first time the effect of nebulized Myo-Ins in patients affected by bronchiectasis [51].

In this study a total of 19 patients, aged between 63 and 73 years old, with bronchiectasis received nebulized Myo-Ins (400 mg in 3 mL 0.9% saline) or placebo for 15 days. All patients treated with nebulized Myo-Ins had a significant decrease in the percentage of solid content in the expectorate (*p* < 0.001) because of a higher hydration of the mucus and a decrease in surfactant tension (*p* < 0.001). Moreover, these modifications also positively correlated with FEV1 (*p* < 0.01) and FEF_25–75%_ (*p* < 0.01) scores [51], evidencing a better lung functionality.

Although this was a pilot retrospective study, it was evident that nebulized Myo-Ins was effective in increasing mucus hydration, restoring mucociliary clearance, and improving lung functionality compared to a placebo group.

Another clinical study tested the action of nebulized Myo-Ins (400 mg in 3 mL 0.9% saline) in 15 patients with different respiratory diseases and symptoms [52]. The population of this study was heterogeneous, in fact, 33.3% of patients had COPD, 20% asthma and recurrent tonsillitis, 13.3% COVID-19, and 6.7% pulmonary emphysema, bronchitis, and otitis. Although they were affected by different respiratory diseases, all of them experienced dyspnea, cough, fever, and general discomfort. Treatment with nebulized Myo-Ins for 15 days was able to significantly improve both SpO_2_ levels (98% (IQR 95–97.5)) and induce a total recovery of all symptoms. In fact, a total of 67% of patients (*p* < 0.001) had a total recovery and improvement of all symptoms [52].

### 3.2. Myo-Inositol and Pulmonary Surfactant

In 1955, Pattle et al. described for the first time the pulmonary surfactant, evidencing its involvement in several lung diseases [53]. Pulmonary surfactant is a surface-active lipoprotein complex lining the pulmonary alveolar surface, synthesized and secreted by type II alveolar epithelial cells (ATII) [54,55]. Its primary function is to reduce alveolar surface tension, thus facilitating breathing and gas exchange, but it also plays an important role in the host defense process [55]. It is predominantly composed of lipids (90%) and 10% protein (SP-A, SP-B, SP-C, and SP-D). Surfactant phospholipids (PLs) account for 80–85% of pulmonary surfactant lipids, including phosphatidylcholine (PC, accounting for about 80%), phosphatidylglycerol (PG, accounting for about 7–15%), and small quantities (accounting for approximately 5% each) of phosphatidylinositol (PI), phosphatidylethanolamine (PE), and phosphatidylserine (PS) [54,55,56].

While SP-B and SP-C are mainly involved in surfactant organization, playing a role in enhancing the surface tension-reducing properties of surfactant [57], SP-A and SP-D participate in pulmonary host defense and modify the innate immune response to clear a variety of bacterial, fungal, and viral pathogens [58,59]. Alterations in the quantity and/or quality of surfactant can lead to important changes in its physiological properties and surface activity, thus leading to serious effects on pulmonary function with the occurrence of several pathological conditions [9].

Neonatal respiratory distress syndrome (RDS) is the most common complication of prematurity leading to significant morbidity in late preterm neonates and even mortality in very low birth weight infants. It is a direct consequence of surfactant deficiency due to either inadequate surfactant production or surfactant inactivation in the context of immature lungs [60].

RDS in preterm infants exhibits incomplete ATII development and insufficient surfactant production, resulting in reduced lung compliance, increased risk of alveolar collapse, difficulty breathing, and impaired gas exchange [61].

The administration of exogenous surfactants to newborn infants with or at risk of RDS was an established and safe practice to compensate the well-documented deficiency of alveolar surfactant by the early 1990s [62].

**Figure 2:**
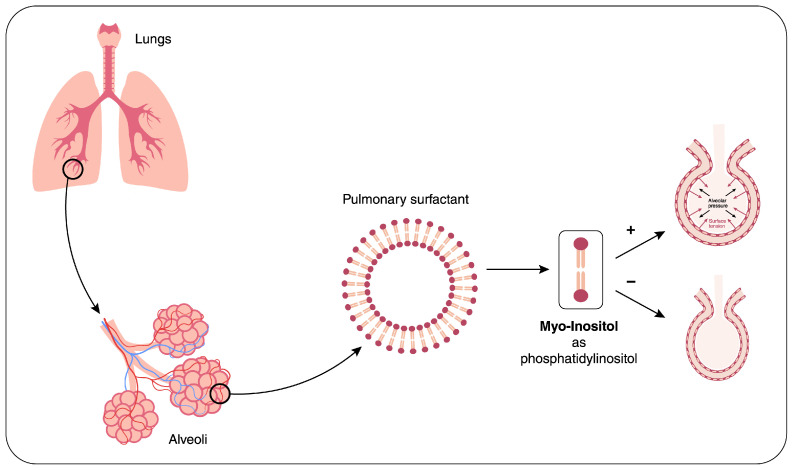
The Pulmonary Surfactant. Myo-Ins is a physiological component of the pulmonary surfactant, in the form of phosphatidylinositol, and contributes to decreased surface tension at the alveolar level.

Myo-Ins is a physiological component of pulmonary surfactant in the form of phosphatidylinositol [63] (Figure 2). Myo-Ins promotes maturation of the surfactant phospholipids, phosphatidylcholine, and phosphatidylinositol. Namely, the synthesis of phosphatidylinositol in type II pneumocytes appears to be dependent on extracellular inositol concentrations [64]. Increased inositol content or inositol-derived phosphatidyl compounds of the surfactant may significantly improve the mechanical properties of alveoli. Inositol and its phosphorylated forms, through their osmolar activity, recruit organic osmolytes and water within the alveolar space [65], thus fostering the reconstitution at the interface of a biofilm layer (featuring a hydrophobic tail and a hydrophilic head), decreasing surface tension, and antagonizing collapsing forces.

Among the most important placebo-controlled trials conducted on this topic, Hallman et al. demonstrated that the administration of intravenous Myo-Ins (80mg/kg/day) to premature infants with RDS receiving parenteral nutrition during the first week of life was associated with increased survival, absence of bronchopulmonary dysplasia, and decreased incidence of retinopathy of prematurity [66].

Infants with RDS may also develop more severe forms of chronic lung disease even after surfactant therapy [67]. By analyzing surfactant composition, differences between premature births with RDS and those with worsening disease emerged: in particular, the latter had very low levels of SP-A protein and phosphatidylinositol [68]. However, intravenous administration of inositol can also correct these shortcomings [66,69].

Surfactant also plays an important role in the upper respiratory tract, both in physiological and pathological contexts, for maintaining and restoring correct mucociliary clearance [70]. Lamellar bodies, which secrete the surfactant, as well as express surfactant apoproteins (SP-A, B, and D), have also been detected at the level of the sinus mucosa [71,72] and biochemical analysis of nasal aspirate of healthy individuals revealed the presence of phospholipids constituting pulmonary surfactant [73]. The comparison between healthy volunteers and volunteers with primary atrophic rhinosinusitis (a condition that involves inflammation of the nasal mucosa, resulting in nasal congestion) showed that in the first group (healthy volunteers), the surfactant phospholipids contained 75.35% phosphatidylcholine, while in the second group (volunteers with primary atrophic rhinosinusitis), the content of total phospholipids was significantly lower, with 41.1% phosphatidylcholine [73]. This evidence shows how the surfactant, in addition to lowering the surface tension at the lung level, also plays an important role in the upper respiratory tract, both in a physiological and pathological context, for the maintenance and restoration of correct mucociliary clearance. For this reason, in recent years, surfactant-based therapy has also become a new and promising approach for treating pathologies of the upper respiratory tract, such as allergic rhinitis and chronic sinusitis [74].

Emerging data have shown that pulmonary surfactant lipids modulate the inflammatory response and antiviral effects in some respiratory viral infections, and pulmonary surfactant lipids have shown promise for therapeutic applications in some respiratory viral infections [56]. Some studies have shown that phosphatidylinositol possesses potent antiviral effects, preventing respiratory syncytial virus (RSV) infection in vivo and in vitro [75]. In another study, intranasal inoculation with phosphatidylinositol reduced the viral load in lungs, eliminated the influx of inflammatory cells, and reduced lung tissue histopathology in RSV-infected mice [76].

### 3.3. Myo-Inositol as a Redox Balancer and Modulator of Inflammation

Inflammation is a biological response of the immune system, a natural defense mechanism that arises in response to a harmful stimulus, such as pathogens, irritants, damaged cells, or radiation [77]. Usually, acute inflammatory responses help to restore an altered equilibrium and homeostasis; unfortunately, when inflammation persists, it becomes chronic, leading to tissue damage and contributing to the development of a large variety of chronic diseases, including respiratory and cardiovascular diseases, atherosclerosis, rheumatoid arthritis, or cancers [78,79].

The production of several cytokines, such as interleukin-1β (IL-1β), tumor necrosis factor-α (TNF-α), interferon-γ (IFN-γ), transforming growth factor-β (TGF-β), and interleukin-8 (IL-8), usually characterizes inflammatory processes [80]. Among them, interleukin-6 (IL-6) has a pivotal role in driving inflammation towards a chronic phase [81]. Its levels are increased in the airway epithelial cells of asthmatic children [82] and in the exhaled air of asthmatic or COPD adult patients [83,84,85]. Moreover, asthmatic and COPD patients exhibit an inverse correlation between levels of IL-6 in the sputum and lung functionality [85,86]. Plasma concentration of IL-6 correlates with systemic inflammation, and it is a marker of poorer outcomes in COPD patients [87]. High levels of IL-6 correlate with respiratory failure and a worse outcome in patients with COVID-19 [88]. Therefore, blocking or targeting the signaling pathway of IL-6 may represent a promising approach to treat and prevent chronic inflammatory diseases [89].

Myo-Ins decreases IL-6 levels in several pathological condition and experimental models. It showed a strong chemo-preventive activity in a KRAS-driven lung cancer model of mice by reducing circulating IL-6 levels and by switching to antitumoral M1 macrophages [90]. Proteomic and cytokine analyses revealed a large reduction in IL-6-related pathways, including STAT3 phosphorylation [90]. Moreover, treatment with Myo-Ins induced a potent reduction in the number, size, and stage of lung premalignant lesions as compared to those raised on control diets [90].

Other studies have proven Myo-Ins’s action in modulating IL-6 levels in other chronic inflammatory diseases such PCOS, obesity, and metabolic syndrome. In this context, Myo-Ins downregulates IL-6 and PI3K (a key factor in the transduction of IL-6 signals), as well as other inflammatory parameters like prostaglandins and cyclooxygenase-2 (COX2) [91,92,93,94].

Oxidative stress has many pathophysiological implications in several airway disorders [95], and it strictly correlates to inflammation [96]. Indeed, inflammatory cells produce several reactive species at the site of inflammation, leading to exaggerated oxidative stress conditions; on the other hand, a plethora of reactive oxygen/nitrogen species can initiate an intracellular signaling cascade that enhances proinflammatory gene expression.

Oxidative stress plays a crucial role in the pathogenesis of asthma [97], in which increased ROS production inversely correlates with FEV_1_ [98]. ROS directly damage biological molecules and lung extracellular matrix, leading to cell dysfunction or death and thus activating the nuclear factor (NF)-kappa B (NF-κB) pathway, which has a central role in regulating the expression of inflammatory genes in airway cells, as happens in asthma and COPD, thus contributing to airway narrowing [99].

In this regard, Myo-Ins could find applications for its antioxidant activities [100,101]. In general, inositol derivatives appear to play a protective role against oxidative stress caused by cell metabolism [102]. Myo-Ins supplementation increases the activity of glucose in the pentose phosphate pathway through an increased production of NADPH—one of the factors that reduce oxidative stress in cells—and it is an essential molecule for the normal functioning of antioxidant cycles [100]. A recent in vivo study demonstrated that Myo-Ins supplementation significantly reduced intracellular levels of ROS in the basal state in endothelial cells obtained from diabetic women [102]. In patients with PCOS, Myo-Ins plus folic acid had beneficial effects on the plasmatic level of total antioxidant capacity (TAC) [103]. In a clinical study on patients with non-alcoholic fatty liver disease (NAFLD), oral pinitol supplementation (an inositol derivate) significantly reduced the content of liver fat and improved glutathione peroxidase (GPx) activity [104]. Another study showed that D-chiro-inositol (DCI) (another stereoisomer of inositol) has an effective role against oxidative stress, as it reduces oxidative stress in the follicular fluid of non-obese PCOS patients. Sixty-eight women, during assisted procreation protocols, reported a significantly higher number of good-quality oocytes (MII) in the DCI group than the untreated control group, with a decrease in oxidative damage in follicular fluid protein markers [105]. In addition, the antioxidant effect of Myo-Ins has been tested to improve sperm quality in human semen samples by reducing in vitro DNA oxidation and thus improving sperm motility and fertility outcomes [106].

Some evidence supports the role of Myo-Ins in directly mitigating key inflammatory pathways in inflammatory lung diseases [107]. In an animal model of ovarian hyperstimulation syndrome (OHSS), a condition that can be characterized by life-threatening events like acute respiratory distress syndrome (ARDS), Myo-Ins significantly reduces several inflammatory signatures, including vascular permeability, VEGF, and COX-2 expressions [108].

## 4. Effect of Myo-Inositol Supplementation in Various Respiratory Diseases

Considering all these general properties, we speculate that Myo-Ins supplementation could be a valid adjuvant therapeutic approach to treat or prevent several respiratory diseases by acting on specific molecular pathways involved in pathologies’ progression (Table 1 and Table 2).

### 4.1. Lung Cancer

Lung cancer is the most common cause of cancer death worldwide. Decreasing the risk of lung cancer or preventing its development in high-risk individuals would be a goal. At a molecular level, the PI3K/PKB/AKT pathway represents a critical pathway constitutively activated in several tumors [115,116], including lung cancer. PI3K/PKB/AKT pathway controls several processes integral in the development of cancer, including protein translation, growth, metabolism, and survival [117].

The clinical importance of Akt activation in lung cancer has been shown by detecting its expression in human bronchial dysplastic lesions and early-stage non-small cell lung cancer specimens, thus conferring a poor prognosis for patients [118]. Agents that affect the PI3K/PKB/AKT pathway may therefore have potential as chemotherapeutic agents, but unfortunately, the harmful side effects of some of them will limit their utility.

In this regard Myo-Ins could be a modulator of tumorigenic lung activities and a well-tolerated inhibitor of the PI3K pathway [90,119]. In vitro studies evidenced that Myo-Ins decreased endogenous levels of Akt phosphorylation in human bronchial epithelial cells in a dose-dependent manner. Also, the activation of extracellular signal-regulated kinase (ERK) was inhibited [111]. ERK activation is observed in 71% of dysplastic lesions, suggesting that it may play a crucial role in lung cancer progression by inhibiting apoptosis of damaged premalignant cells that could ultimately undergo full transformation. Myo-Ins treatment significantly decreased both phospho-Akt and phospho-ERK expression levels in dysplastic lesions of heavy smokers (*p* = 0.001 and 0.01, respectively) [111].

In several studies, oral Myo-Ins inhibited lung tumorigenesis in mice exposed to carcinogens [112,120,121]. Enstensen et al. [122] showed that mice fed with a 3% Myo-Ins diet had 40% fewer lung adenomas than the control, and when Myo-Ins was added to dexamethasone, an additive effect on the inhibition of pulmonary adenoma formation also occurred [122].

The same results were obtained in other vivo studies [90,120,123,124].

The results obtained in animal studies led to studies in humans. A phase I study for lung cancer chemoprevention showed that Myo-Ins supplementation should be investigated as a chemopreventive agent against lung cancer. In smokers with bronchial dysplasia, Myo-Ins supplementation, in a daily dose of 18 g per os, induced a significant regression of individual pulmonary dysplastic lesions (91% in Myo-Ins group versus 48% in placebo group) [109].

Unfortunately, a randomized phase IIb trial did not obtain the same results, probably due to inadequate sample size. However, a significant reduction of the IL-6 levels in BAL and a significant decrease in the gene expression signature reflective of PI3K activation within the cytologically normal bronchial airway epithelium were seen among complete responders in the Myo-Ins group [110]. It is well known that persistent inflammation plays a critical role in carcinogenesis, therefore multiple anti-inflammatory agents have been investigated for the chemoprevention of lung cancer, such as non-steroidal anti-inflammatory drugs (NSAIDs). Their principal therapeutic effect is based on the inhibition of the cyclooxygenase activity of prostaglandin-endoperoxide synthase (PTGS) 1 and 2 and the consequent suppression of the formation of arachidonic acid (AA) metabolites [125].

Unfortunately, NSAIDs can induce serious side-effects, particularly after long-term use, in the elderly, and in subjects with other comorbidities [126]. Myo-Ins inhibits COX-2 activity, as evidenced in several other models [127,128]. Although the exact mechanism involved in this inhibition remains unknown, and there are no studies on respiratory models, a proposed hypothesis states that Myo-Ins may directly interact with COX-2 through a deep hydrophobic channel in the active site, involving polar and non-polar interactions [129]. This hypothesis and the scientific evidence in other models would support the use of Myo-Ins in this context. Moreover, Myo-Ins is considered as GRAS by the FDA, and its supplementation is applicable for a long period, without adverse events.

### 4.2. Asthma

Asthma is among the most common chronic diseases in the world, affecting more than 300 million people. It is a disease characterized by airway inflammation, elevated mucus production, and airway obstruction [130]. Increased deposition of ECM proteins in the reticular basement membrane region, lamina propria, and submucosa is characteristic of asthmatic airways, thus contributing to airway wall thickening and airflow obstruction. Fibroblasts are the major producer of ECM. Asthmatic airway epithelial cells stimulate lung fibroblasts to produce collagens, fibronectin, and TGF-β1. TGF-β1 is strongly implicated in airway remodeling and is released by eosinophils at the site of allergic inflammation. Moreover, it promotes metalloproteinase-9 (MMP-9) production, found in BAL fluid as well as plasma from asthmatics [131].

Another distinct hallmark of airway remodeling in asthma is subepithelial fibrosis that is primarily mediated by submucosal resident fibroblasts that proliferate and differentiate into myofibroblasts. Increased numbers of myofibroblasts are present in the airway walls of asthmatic individuals compared to controls [132].

Inefficient autophagy has been demonstrated to be related to myofibroblast differentiation and fibrosis in various tissues, including the lung. Autophagy suppression may occur in response to TGF-β1. Specifically, TGF-β1 activates the PI3K/Akt/mTOR pathway which inhibits the initiation of autophagosome formation [133]. It is now well recognized that resident airway smooth muscle (ASM) cells and fibroblasts drive key cellular and structural features of asthmatic airway remodeling, specifically the increase in ASM mass and subepithelial fibrosis [134].

To the best of our knowledge Myo-Ins still has not been used for treating asthma diseases but, based on scientific evidence from other respiratory diseases, we speculate that it could be used for different purposes.

It could modulate inflammation by acting on NF-κB and reducing the proinflammatory cytokines (IL-6, IL-8, and TNF-α) involved [135]; it could act directly on the PI3K/Akt pathway, involved in airway remodeling and survival of activated immune cells [136]; it could modulate EMT by inhibiting TGF-β1 activity, thus preventing airway remodeling associated to chronic asthma [137]. The accumulation of mucus in the airways in asthma is well recognized. It is associated with hypertrophy and hyperplasia of epithelial goblet cells and hypertrophy of submucosal glands. Impaired clearance of mucus is present during exacerbations of asthma. The rheological properties of mucus are altered in asthma which may lead to reduced clearance and accumulation of mucus within the airways [138]. Myo-Ins, being a potent osmolyte, could increase the hydration of mucus, thus favoring mucociliary clearance, as already evidenced in patients with bronchiectasis [51].

### 4.3. Chronic Obstructive Pulmonary Disease (COPD)

COPD represents the third cause of death after cardiovascular disease and stroke, the fifth cause of chronic disability, and a leading cause of emergency hospital admission worldwide [139]. COPD is a heterogeneous lung disease characterized by a variety of chronic respiratory symptoms and arises from the complex interplay of multiple factors. Given that abnormal activation of the PI3K/AKT pathway is crucial in COPD progression, inhibiting the PI3K/Akt signaling pathway, to reduce inflammation, apoptosis, and oxidative stress in cells, could represent a crucial step for COPD treatment [140]. Unfortunately, studies with Myo-Ins treatment on COPD models are still lacking. However, the results obtained in other models, concerning the inhibition of the PI3K/AKT pathway, let us assume that, Myo-Ins could act as an effective adjuvant approach.

COPD is associated with chronic inflammation of the lung, which particularly affects peripheral airways and the lung parenchyma and leads to small airway fibrosis and emphysema which are progressive [141]. Also, NF-κB is hyperactivated in COPD, inducing an increased production of proinflammatory cytokines. Myo-Ins could modulate the production of inflammatory cytokines, such as TNF-α, IL-6, and IL-8, through the regulation of pathways such as NF-κB and PI3K/AKT.

EMT contributes to airway remodeling and fibrosis in COPD [142]. Moreover, EMT has been regarded with interest as the origin of lung cancer among COPD patients. Cigarette smoke and oxidative stress cause damage to epithelial cells, leading to apoptosis and emphysema and, the expression of HIF-1 and vascular endothelial growth factor (VEGF), accelerating the proliferation and invasion of tumors.

Myo-Ins, by inhibiting the TGF-β pathway (a key driver of EMT), as already evidenced in other tumor models [143], could limit the transformation of epithelial cells into fibroblasts and reduce the deposition of ECM and prevent lung cancer development.

COPD mucus can accumulate in the airway, causing narrowing of the lumen, and this accumulation has been correlated with the degree of airflow obstruction. Cigarette smoking has been associated with changes in the epithelium, including goblet cell hyperplasia and metaplasia and inflammation of the submucosal mucous glands [138]. Therefore, Myo-Ins, as already evidenced in patients with bronchiectasis, which may co-exist as an overlap syndrome with COPD (BCOS), could improve mucociliary clearance [144].

### 4.4. Idiopathic Pulmonary Fibrosis (IPF)

Idiopathic pulmonary fibrosis (IPF) is a progressive lung disease with high mortality and limited treatment options [145].

The pathogenesis of IPF has not been clarified, and its treatment is limited to two medications, pirfenidone and nintedanib, which only delay the decline of lung function, without preventing mortality and significantly reducing fibrosis.

In general, fibrosis is defined as a pathologic deposition of ECM during wound healing. Initiation of the wound healing process elicits an inflammatory response that ultimately recruits fibroblasts and activates myofibroblasts to deposit ECM in the form of collagen and other proteins. While wound healing typically resolves with apoptosis of myofibroblasts, in fibrotic disease states, there is persistence of profibrotic activators and myofibroblasts [145].

Irreversible matrix deposition and pulmonary remodeling characterize IPF [146].

Recently, the ability of inositol supplementation as an antifibrotic therapy has been tested in a bleomycin-induced pulmonary fibrosis mouse model [130]. Moreover, bleomycin-exposed mice treated with Myo-Ins had a significant improvement in histologic fibrosis and collagen deposition [130].

Researchers have also found that IPF patients have a particular metabolic deficiency in arginosuccinate synthase 1 (ASS1). ASS1 is a rate-limiting enzyme in de novo biosynthesis of arginine (Arg) [113], a semi essential amino acid in adults and rodents, that participates in multiple cellular functions (cell division, ammonia removal, nitric oxide synthesis, glycogenesis, protein synthesis, and synthesis of collagen). The precise role of Arg in IPF remains controversial, but evidence indicates that its metabolism is altered in lung fibrosis, predisposing patients to abnormal collagen synthesis and dysregulated airway remodeling [147].

IPF fibroblasts with ASS1 deficiency display a downregulated inositol level and upregulated expression of several enzymes (i.e., MIOX) involved in inositol catabolism and phosphatidylinositol metabolism, suggesting that ASS1-deficient lung fibroblasts tend to consume and/or eliminate most cellular inositol [113]. Myo-Ins treatment not only inhibited fibrotic molecules (e.g., COL1A1 and α-SMA) but also repressed cell invasiveness in IP fibroblasts, a pathological feature of EMT [113].

Although there are no other studies concerning Myo-Ins and the treatment of IPF, these results seem to be very promising and would merit being deepened with further investigations as they indicate the possibility of inositol supplementation as a viable adjuvant antifibrotic therapeutic strategy for IPF.

### 4.5. Acute Respiratory Distress Syndrome (ARDS)

Acute respiratory distress syndrome (ARDS) is a devastating inflammatory lung disease characterized by dysregulated inflammation and alveolar–capillary barrier disruption. Despite progress in anti-inflammatory drugs and assisted mechanical ventilation, the mortality rate of severe ARDS is still high [148]. Although there have been improvements in supportive measures, such as lung-protective ventilation and fluid management strategies, there is still a lack of targeted treatments to improve clinical outcomes. EMT and the fibrosis process play an important role in the development of ARDS. An impaired autophagy has also been observed in models of fibrosis and may represent a pathogenic feature for development in the case of ARDS. Autophagy is a cellular homeostatic program that governs the turnover of long-lived proteins and dysfunctional organelles via sequestration in double-membrane-bound autophagosomes and subsequent lysosome-dependent degradation [149]. Recently an in vivo ARDS mouse model was used to test the efficacy of Myo-Ins in counteracting ARDS [114]. Results were obtained in an in vivo model and confirmed in vitro, evidencing that Myo-Ins treatment reduced proinflammatory markers (IL-1β, IL-6, IL-17, MCP-1), increased autophagy, and inhibited EMT by downregulating SLUG expression, an EMT regulator in various diseases and lung cancer [150].

## 5. Conclusions

Respiratory diseases include a plethora of pathological conditions affecting the upper and the lower respiratory system. They may vary from mild and self-limiting conditions to life-threatening and persistent diseases, and represent the third cause of death worldwide. The scientific evidence showed that Myo-Ins, a component of pulmonary surfactant, has a wide spectrum of properties which can be effective for the management of different respiratory diseases. To date, only sporadic clinical trials have tested its efficacy on respiratory diseases. Hopefully, future randomized clinical trials will increase knowledge of its beneficial effects and support, its role as an adjuvant therapeutic strategy in the management of these disorders. 

## Figures and Tables

**Table 1:** Effect of Myo-Inositol (Myo-Ins) supplementation on different respiratory disease models.

Model	Diseases	Routes of Administration	Effect	Ref.
Humans	Bronchiectasis	Nebulized (400 mg/3 mL 0.9%)	Increased MCC; improvement of lung function; decrease in surface tension	[51]
Humans	COPD, COVID-19, asthma, pulmonary emphysema, bronchitis, otitis	Nebulized (400 mg/3 mL 0.9%)	Increase in SpO2; resolution of respiratory symptoms (dyspnea, cough, phlegm production)	[52]
Humans	Neonatal RDS	Intravenous (80 mg/kg/day)	Increase in survival, absence of bronchopulmonary dysplasia, and decreased incidence of retinopathy of prematurity	[66]
Humans	Heavy smokers at risk of lung cancer	Oral (18 g/d)	Regression of preexisting dysplastic lesions	[109]
Humans	Heavy smokers at risk of lung cancer	Oral (9 g/once/day for 2 weeks and then twice/day for 6 months)	Reduction of the IL-6 levels in BAL and a decrease in PI3K activity	[110]
Humans	Smokers’ bronchial biopsies	-	Decrease in endogenous and tobacco-carcinogen-induced activation of AKT and ERK	[111]
Mice	Lung cancer	3 g of diet per mouse per day	Reduction of IL-6 levels and switching to antitumoral M1 macrophages; potent reduction in the number, size, and stage of premalignant lesions as compared to those raised on control diets	[90]
Mice	Lung cancer	1% added to diet	Reduction in tumor formation	[112]
Mice	IPF	2.4 g/kg	Reduction of fibrosis and cell invasiveness	[113]
Mice	ARDS	60 mg/kg	Inhibition of EMT (SLUG); increased autophagy; inhibition of proinflammatory markers (IL-1β, IL-6, IL-17, MCP-1)	[114]

Abbreviations: MCC—mucociliary clearance; COPD—chronic obstructive pulmonary disease; SpO_2_—oxygen saturation; RDS—respiratory distress syndrome; ARDS—acute respiratory distress syndrome.

**Table 2:** Effect of Myo-Inositol supplementation on various respiratory diseases.

**Pulmonary diseases**	Effect of Myo-Inositol supplementation
**Lung cancer**	Inhibition of PI3K/(PKB/AKT) pathway; inhibition of ERK pathway; inhibition of COX2 activity
**ARDS**	Increasing autophagy; inhibition of EMT (upregulation of E-cadherin; downregulation of SLUG and N-cadherin); downregulation of fibrosis and HIF-1α
**IPF**	Downregulation of fibrosis and EMT
**Asthma**	Modulation of inflammation, by acting on NF-κB, reducing proinflammatory cytokines (IL-6, IL-8, and TNF-α); modulation of PI3K/Akt pathway; modulation of EMT by inhibiting TGF-β1 activity; reducing airway and bronchial remodeling that accompanies chronic asthma
**COPD**	Inhibition of PI3K/(PKB/AKT) pathway; modulation of inflammatory cytokines, such as TNF-α, IL-6, and IL-8; increased mucociliary clearance

Abbreviations: COPD—chronic obstructive pulmonary disease; ARDS—acute respiratory distress syndrome; IPF—idiopathic pulmonary fibrosis; EMT—epithelial–mesenchymal transition; COX2—cyclooxygenase-2; HIF-1α—hypoxia-inducible factor alpha; PI3K/Akt—phosphatidylinositol 3-kinase (PI3K)/protein kinase B (PKB/Akt) signaling; TGF-β1—transforming growth factor beta 1; TNF-α—tumor necrosis factor alpha.

## Data Availability

Not applicable.
